# Endoscopic Ex Vivo Evaluation of Bile Concentrations by Narrow Band Imaging: A Pilot Study

**DOI:** 10.1155/2015/367848

**Published:** 2015-05-13

**Authors:** Roberta Maselli, Haruhiro Inoue, Haruo Ikeda, Manabu Onimaru, Akira Yoshida, Esperanza Grace Santi, Hiroki Sato, Nikolas Eleftheriadis, Shin-ei Kudo

**Affiliations:** ^1^Digestive Disease Center, Showa University Northern Yokohama Hospital, 35-1 Chigasakichuo, Tsuzuki-ku, Yokohama 224-8503, Japan; ^2^Surgical Sciences Department, Umberto I° General Hospital, Sapienza University of Rome, Rome, Italy; ^3^De La Salle University Medical Center, Dasmarinas, Philippines

## Abstract

*Background*. Bile juice plays a major role in duodenogastroesophageal reflux (DGERD). Several devices to directly measure the bile concentration have been proposed. We aimed to ex-vivo evaluate the bile concentration by narrow band imaging (NBI). *Method*. From six surgical cholecystectomies, the content of the gallbladders was aspirated and the total biliary acid (TBA) concentration was evaluated. 2 mL was employed for serial twofold dilutions. Each dilution was scoped. Images on white light (WL) and NBI were captured and grouped accordingly to NBI-appearance and TBA-concentration. *Results*. Nondiluted bile had a TBA-concentration of 61965 ± 32989 *μ*mol/L. Final dilution (1 : 4096) had 1.16 *μ*mol/L. NBI and correspondent WL images were grouped into seven groups, and an NBI/Bile scale was created. *Conclusion*. The scale showed that not only NBI scale but also white light scale could be useful to predict the bile concentration. This initial study shows that NBI has a potential role in the detection of DGERD and further investigation is warranted to distinguish the presence and the concentration of bile, especially at very low TBA concentrations.

## 1. Introduction

In the pathogenesis of reflux esophagitis, acids, bile acids, and pepsin play a major role in damaging the esophageal epithelium [1–3]. However, the mechanisms by which the luminal contents affect the barrier function of the stratified epithelial cell layers remain unclear.

Duodenogastroesophageal reflux (DGER) of bile acids has been hypothesized to play an important role in the development of oesophageal symptoms, oesophagitis, Barrett's esophagus (BE), and oesophageal adenocarcinoma [4]. This is supported in animal models in which oesophageal bile exposure (with or without acid or pancreatic juice) by either direct perfusion or total gastrectomy with oesophagojejunal anastamosis results in severe oesophagitis, Barrett's metaplasia, and adenocarcinoma [5].

It has also been proposed that bile reflux into the airway contributes to extraoesophageal GERD manifestations, although the evidence to support this contention is largely indirect.

The first report on narrow band imaging was published more 19 years ago [6] to demonstrate the contrast between the vascular pattern and the adjacent mucosa of the underside of the human tongue using five narrow band illuminations and three broadband illuminations.

**Figure 1:**
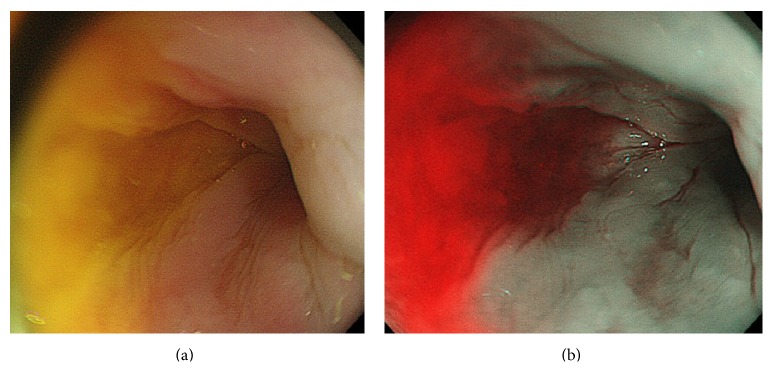
DGERD during esophagoscopy; note how severe the bile reflux appears on NBI (b) compared with correspondent white light image (a).

The technology consists of placing narrow band pass filters in front of a conventional white light source to obtain tissue illumination at selected narrow wavelength bands. These bands produce the greatest contrast between vascular structures and the surrounding mucosa. Currently available NBI systems use 2 narrow band filters that provide tissue illumination in the blue (415 nm), corresponding to the main peak on the absorption spectrum of hemoglobin, and green (540 nm) spectrum of light, corresponding to a secondary hemoglobin absorption peak. NBI generates a darker field of view than its white light counterpart. Moreover, the presence of bile and blood (e.g., after biopsy) obscures the view under NBI because these fluids strongly absorb the narrow band light [7]. The NBI red coloration of the yellowish organic fluids (bile, intestinal residues) is a common experience for endoscopists. More often, because of a normal low contrast between the bile and the pinkish esophageal mucosa, when switching to NBI the reddish bile reflux seems more severe than the one seen with white light (Figure 1). On the other hand, whether NBI is useful for DGER diagnosis and severity assessment has never been investigated. NBI therefore has a potential to allow a one-step, less invasive DGERD diagnostic tool.

The interaction of tissue structures with light, and consequently the appearance of the structures, is wavelength dependent. The different coloration of mucosal features with NBI is achieved by observation of light transmission at selected wavelengths (or colors).

Aim of this pilot study was to evaluate the utility of NBI to distinguish and categorize different concentration on bile in an in vitro model.

## 2. Materials and Methods

From six consecutive routinely performed surgical cholecystectomies, the entire content of the removed gallbladders was aspirated. Part of the bile juice was sent to the laboratory to evaluate the total biliary acid (TBA) concentration. TBA concentration was evaluated by a quantitative test (Modular P800, Hoffmann-La Roche Ltd., Basel, Switzerland); it was performed three times for each sample and finally expressed as *μ*mol/L. Meanwhile, 2 mL of the bile was employed for serial twofold dilutions using sterile water as solvent. The dilutions achieved were then stored in white plastic test tube. Each test tube was finally scoped with the GIFH260 (Olympus Medical Systems Co. Tokyo, Japan), and images on white light and NBI were captured and stored in a dedicated software (SolemioENDO ProStudy; Olympus Optical Co Ltd., Tokyo, Japan). Stored images were then grouped accordingly to the NBI reddish appearance and TBA concentration. Corresponding white light images were grouped accordingly. TBA concentration of each group was calculated and shown as mean ± standard deviation.

## 3. Results

**Figure 2:**
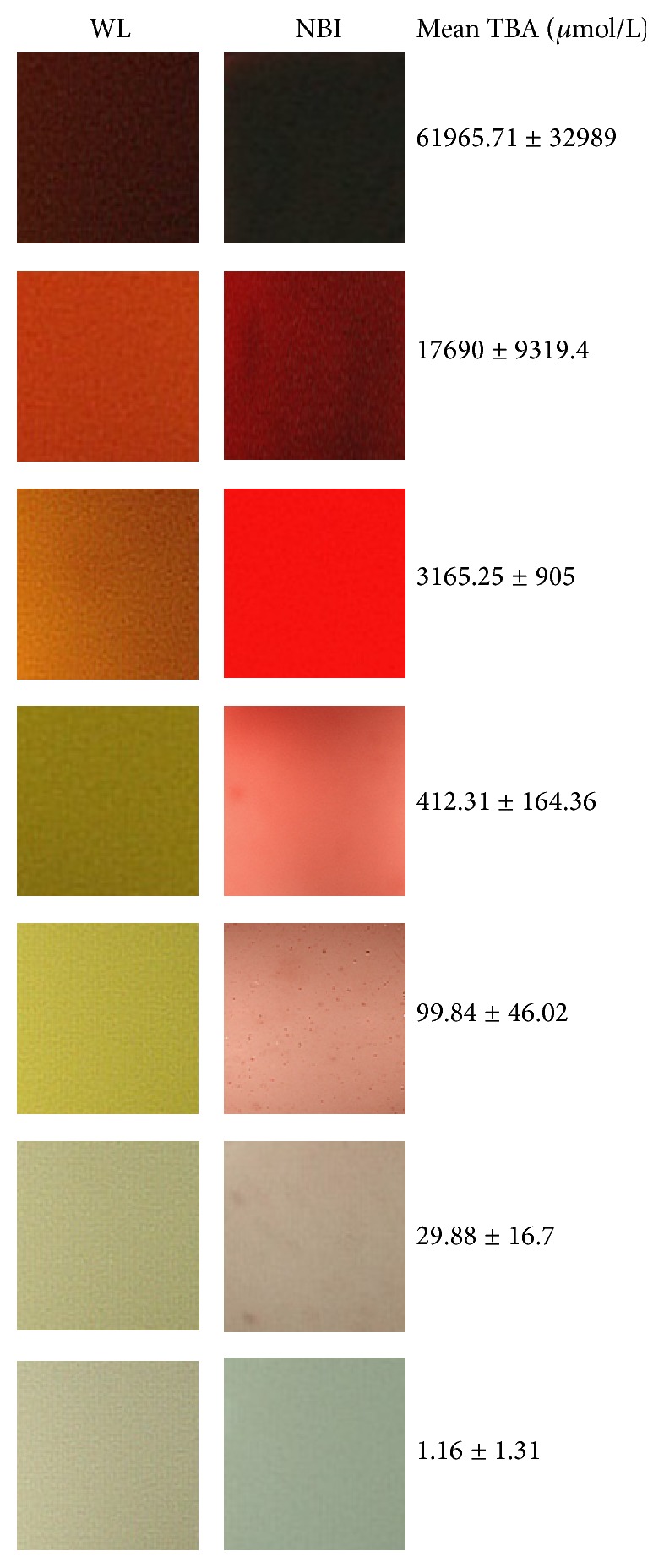
Bile concentration scale on white light (WL) and NBI. Each correspondent mean TBA concentration is reported on the right.

Nondiluted bile from the gallbladders had a mean TBA concentration of 61965 ± 32989 *μ*mol/L (Group 1). It was possible to group NBI images into seven different groups according to the color intensity. A bile concentration scale was created, as showed in Figure 2. Sequential twofold dilutions had the following TBA concentration: Group 2 (1 : 4 dilution) had 17690 *μ*mol/L, Group 3 (1 : 16 dilution) had 3165.25 *μ*mol/L, Group 4 (1 : 64 dilution) had 412.31 *μ*mol/L, Group 5 (1 : 256 dilution) had 99.84 *μ*mol/L, and Group 6 (1 : 024 dilution) had 29.88 *μ*mol/L. Final dilution (1 : 4096) had mean 1.16 ± 1.31 *μ*mol/L (Group 7).

## 4. Discussion

Bile acids can stimulate esophageal squamous epithelial cells and Barrett's epithelial cells to produce substances that might promote esophageal inflammation (e.g., IL-8 and COX-2) and bile acids can cause oxidative stress and DNA damage. Bile acids can induce esophageal squamous cells to change their gene expression patterns to resemble intestinal cells and can cause Barrett's epithelial cells to increase their expression of intestinal-type genes [8].

These data suggest that in patients with GERD the bile acids may contribute to the pathogenesis of symptoms, esophagitis, Barrett's metaplasia, and Barrett's related carcinogenesis [9].

Historically, measurement of DGER has been thought to be feasible by scintigraphy, endoscopic gastric aspiration techniques [10], ambulatory gastric pH monitoring [11], and more recently by endoluminal spectrophotometric techniques (Bilitec) and impedance monitoring. Bilitec 2000 is currently the only commercially available device that is proven effective in measuring bile reflux. Bilitec 2000 has shown that patients with severe esophagitis [12] and patients with BE have more bilirubin reflux episodes than normal subjects and that these reflux episodes coincide with acid reflux episodes. Bilitec is an external probe to be inserted in the esophagus. In contrast to Bilitec, NBI is incorporated in the endoscope activated by a push of a button.

Considering the created scale, it seems that not only NBI scale but also white light scale could be useful to predict the bile concentration. However, this is an in vitro study and the contrast between the bile and the physiologic color of the mucosa must be taken into consideration and checked in the future. The bile concentrations were stored and scoped in a white plastic tube and this white background color could interfere with the NBI/white images. At the same time, these tubes were chosen to have the same background, being impossible to reproduce the real in vivo conditions of normal gut.

Although future in vivo studies are needed to confirm this bile scale, this initial study shows that NBI has a potential role in the detection of DGERD and further investigation is warranted to distinguish the presence and the concentration of bile, especially at very low TBA concentrations.
